# Design, Synthesis, and Pharmacology of New Triazole-Containing Quinolinones as CNS Active Agents

**DOI:** 10.3390/molecules28041987

**Published:** 2023-02-20

**Authors:** Wennan Zhao, Mingxia Song, Yi Hua, Yangnv Zhu, Wenli Liu, Qishan Xia, Xianqing Deng, Yushan Huang

**Affiliations:** 1 Health Science Center, Jinggangshan University, Ji’an 343009, China; 2 Ji’an Key Laboratory of Personalized Drugs for Neuropsychiatric Diseases, Ji’an 343009, China; 3 Center for Evidence Based Medical and Clinical Research, First Affiliated Hospital of Gannan Medical University, Ganzhou 341000, China

**Keywords:** quinolinone, triazole, antidepressant, antiseizure, anticonvulsant, forced swim test, GABA, maximal electroshock seizure

## Abstract

Epilepsy and major depressive disorder are the two of the most common central nervous system (CNS) diseases. Clinicians and patients call for new antidepressants, antiseizure medicines, and in particular drugs for depression and epilepsy comorbidities. In this work, a dozen new triazole-quinolinones were designed, synthesized, and investigated as CNS active agents. All compounds reduced the immobility time significantly during the forced swim test (FST) in mice at the dosage of 50 mg/kg. Compounds **3f**–**3j** gave superior performance over fluoxetine in the FST with more reductions of the immobility time. Compound **3g** also reduced immobility time significantly in a tail suspension test (TST) at the dosage of 50 mg/kg, though its anti-immobility activity was inferior to that of fluoxetine. An open field test was carried out and it eliminated the false-positive possibility of **3g** in the FST and TST, which complementarily supported the antidepressant activity of **3g**. We also found that almost all compounds except **3k** exhibited antiseizure activity in the maximal electroshock seizure (MES) model at 100 or 300 mg/kg. Compounds **3c**, **3f**, and **3g** displayed the ED_50_ of 63.4, 78.9, and 84.9 mg/kg, and TD_50_ of 264.1, 253.5, and 439.9 mg/kg, respectively. ELISA assays proved that the mechanism for the antiseizure and antidepressant activities of compound **3g** was via affecting the concentration of GABA in mice brain. The molecular docking study showed a good interaction between **3g** and the amino acid residue of the GABA_A_ receptor. Excellent drug-like properties and pharmacokinetic properties of compound **3a**–**l** were also predicted by Discovery Studio. These findings provided a new skeleton to develop agents for the treatment of epilepsy and depression comorbidities.

## 1. Introduction

Epilepsy and major depressive disorder are two of the most common central nervous system (CNS) diseases worldwide [1,2]. According to the data provided by the World Health Organization (WHO), the estimated incidence of depression is about 4.4% globally, which means that more than 350 million people are now living with depression [1]. The WHO listed depression as the top cause of disability in the world (responsible for 7.5% of disabled patients in 2015), and is also the leading cause of suicide, with nearly 800,000 per year. Meanwhile, more than 50 million people worldwide are struggling with epilepsy. It causes more than 125,000 deaths per year. According to the *Global Burden of Epilepsy Report*, 13 million disability-adjusted life years were contributed by epilepsy per year [2].

During the last 20 years, depression was frequently found as a clinical co-morbidity or sequel to epilepsy and anxiety [3,4,5]. The common pathological basis of them has been found successively [6,7,8,9]. Clinical studies have found that patients with epilepsy (PWE) are more likely to also suffer from depression [10]. A community-based epidemiologic study estimated that one-third of PWE will develop depressive symptoms in their lifetime [11].

The high frequency of this comorbidity requires early diagnosis and pharmacotherapy, which presented clinicians with an arduous task: how to prescribe the patients with epilepsy and depression comorbidities. The relatively high proconvulsant risk of tricyclic antidepressants (TCAs) prevents their use in PWE [12,13]. The majority of reports and research have supported the choice of selective serotonin reuptake inhibitors (SSRIs) and serotonin noradrenaline reuptake inhibitors (SNRIs) as the first-line drugs for patients with this comorbidity. However, the side effects of SNRIs and SSRIs, including anxiety, agitation, gastrointestinal symptoms, changes in appetite and weight, and sexual disturbances limit their application for this comorbidity. SSRIs can aggravate the weight gain induced by antiseizure medicines (ASMs), including, for instance, carbamazepine, pregabalin, valproic acid, and gabapentin. All SNRIs and SSRIs can cause sexual disturbances, which are also relatively common among PWE. SSRIs can also induce osteoporosis and/or osteopenia, which could inhibit the therapeutic effect of enzyme-inducing ASMs [14,15,16]. In summary, the combination of available antidepressants and ASMs is not an ideal choice for this comorbidity considering the existing and potential side effects and drug-drug interactions [17,18,19,20]. Based on the above, it is valuable and urgent to find effective and low-toxic agents for patients with this comorbidity.

Quinolinone and its derivatives comprise an important group of heterocyclic compounds that exhibit a wide range of pharmacological properties such as anticonvulsant [21], anti-cancer [22], antifungal, anti-inflammatory [23], and antidepressant activities [24]. Persistent efforts have been made over the years to develop novel congeners with superior biological activities and the minimal potential for undesirable side effects. Earlier studies by Oshiro et al. [25] have demonstrated that 3,4-dihydro-2(1H)-quinolinones have promising antidepressant activities. Several studies in this area have confirmed the antidepressant properties of aripiprazole, which is a 3,4-dihydro-2(1H)-quinolinone-containing compound [26,27,28].

**Figure 1:**
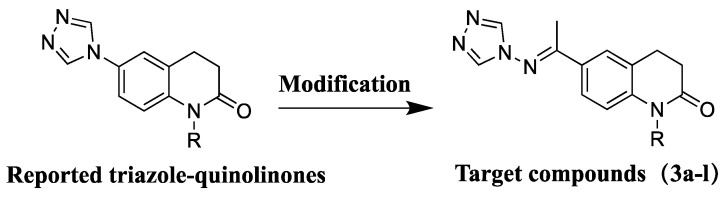
The modification of the leads to the targets (**3a**–**l**).

We prepared numerous triazole-quinolinones and evaluated their antidepressive and antiseizure effects as new CNS agents in our previous report (Figure 1) [29]. Most compounds showed antiseizure action in the maximal electroshock seizure (MES) model at the dosage of 100 or 300 mg/kg. Compounds **5i** (R = CH_2_C_6_H_4_(*o*-F)), **5j** (R = CH_2_C_6_H_4_(*m*-F)), **5m** (R = CH_2_C_6_H_4_(*m*-Cl)), and **5n** (R = CH_2_C_6_H_4_(*p*-Cl)) showed potent antidepressant activity in the forced swim test (FST). It is interesting to find that compounds **5i** and **5m** exhibited antidepressant and antiseizure effects simultaneously. Unfortunately, their antiseizure activity was just found at the maximum dose applied (300 mg/kg). Their antiseizure activity needs to be improved further.

Aiming to search for and obtain new molecules with higher antiseizure and antidepressive activities, in this work, some new derivatives were designed using the triazolequinolinones as leading compounds (Figure 1). An imide group was inserted between triazole and quinolinone by using the vinylogy principle, which will keep the core pharmacophores and electron distribution, but alter the distance from triazole to quinolinone. Herein, the synthesis and pharmacological screening of 12 new triazole-quinolinone derivatives (**3a**–**l**) were described for their antidepressant and antiseizure.

For drugs of the CNS, their neurotoxicity is unavoidable. The CNS drugs, especially antiseizure drugs and antidepressants, have different degrees of neurotoxicity. Therefore, a rotarod test was carried out in order to evaluate the neurotoxicity of the synthetic compounds. As described above, a common pathological basis of epilepsy and depression has been found. Some neurotransmitters such as GABA and 5-HT are associated with their common pathogenesis. Therefore, some tests related to the GABA and 5-HT were also undertaken to explore their possible mechanisms of action.

## 2. Results and Discussion

### 2.1. Chemistry

**Scheme 1:**
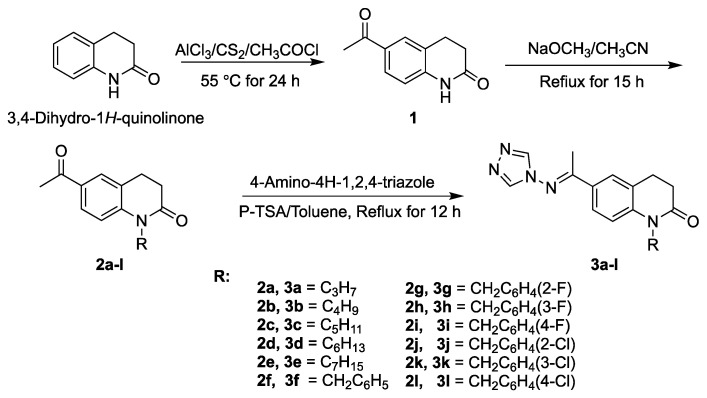
The synthetic route of target compounds **3a**–**l**.

According to the route depicted in Scheme 1, the target compounds **3a**–**l** were prepared. First, 3,4-dihydro-2(1*H*)-quinolinone (**1**) was acetylated with acetyl chloride in CS_2_ using AlCl_3_ as Lewis acid to give 6-acetyl-3,4-dihydroquinolin-2(1*H*)-one (**1**) [30]. The intermediate **1** was alkylated by haloalkanes in the presence of NaOCH_3_ to provide the intermediates **2a**–**l** [29]. Finally, compounds **3a**–**l** were synthesized by the condensation of compounds **2a**–**l** with 4*H*-1,2,4-triazol-4-amine under the catalytic condition of PTSA in refluxed toluene. ^1^H and ^13^C Nuclear Magnetic Resonance (NMR) spectroscopy, as well as high-resolution mass spectroscopy (HR-MS), were conducted for all the target compounds to characterize their structures.

Take compound **3a** as an example in the structure confirmation. In the ^1^H-NMR spectrum, the absorption peak of CH_3_ in the propyl group was found to be 0.98 ppm as a triplet. The absorption peaks of CH_2_ in the propyl group were found at 1.64–1.62 ppm. Another CH_2_ in the propyl group was found at 3.94 ppm. A singlet due to CH_3_ was observed at 2.37 ppm. Two triplets due to CH_2_ in the quinoline ring was observed at 2.70 and 2.98 ppm, respectively. Three aromatic hydrogens on the benzene ring gave the absorption peak at 7.01 and 7.78–7.81 ppm. Two hydrogens from triazole gave the singlet at 8.24 ppm due to the symmetry. The absorption peak in the hydrogen spectrum of compounds **3a** is completely in conformity with the hydrogen signal in the structure. The ^13^C-NMR spectra also gave accurate information about the structure of the compounds **3a**, which involved 15 kinds of carbon in different chemical environments. Moreover, the HR-MS of **3a** displayed an [M + H]^+^ signal at m/z 298.1659, which corresponded to its molecular weight of 298.1662.

### 2.2. Antidepressant Activities

**Figure 2:**
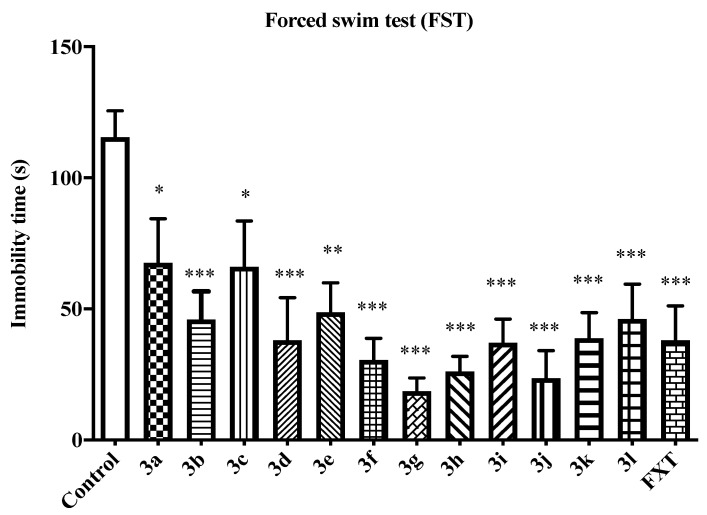
Effects of compounds **3a**–**l** and FXT (i.p., 50 mg/kg) on the duration of immobility time in the FST. Ten animals were used for each group. * *p* < 0.05 compared to the control group, ** *p* < 0.01 compared to the control group, *** *p* < 0.001 compared to the control group.

After intraperitoneal administration (i.p.) of 50 mg/kg, the antidepressive effect of compounds **3a**–**l** were evaluated using FST. Fluoxetine (FXT), as a representative drug of the SSRIs, was used as the positive control with the same dosage (i.p., 50 mg/kg). The FST, as a classic animal model imitating a depressive environment [31], is one of the most commonly used antidepressant screening models due to its low cost, high efficiency, and reliability [32,33,34]. It can be considered that a compound has an antidepressant activity if it can decrease the immobility time of mice in FST. As present in Figure 2, all compounds could shorten the immobility time of mice effectively, which indicated their potent antidepressant activities. Compounds **3b**, **3d**, and **3f**–**3l** showed better performance in reducing immobility time with *p* < 0.001 compared with the control group. Fluoxetine also contributed to a significant reduction for the immobility time with *p* < 0.001. In particular, compounds **3f**–**3j** exhibited more reductions in the immobility time than fluoxetine, although there was no statistical difference.

**Figure 3:**
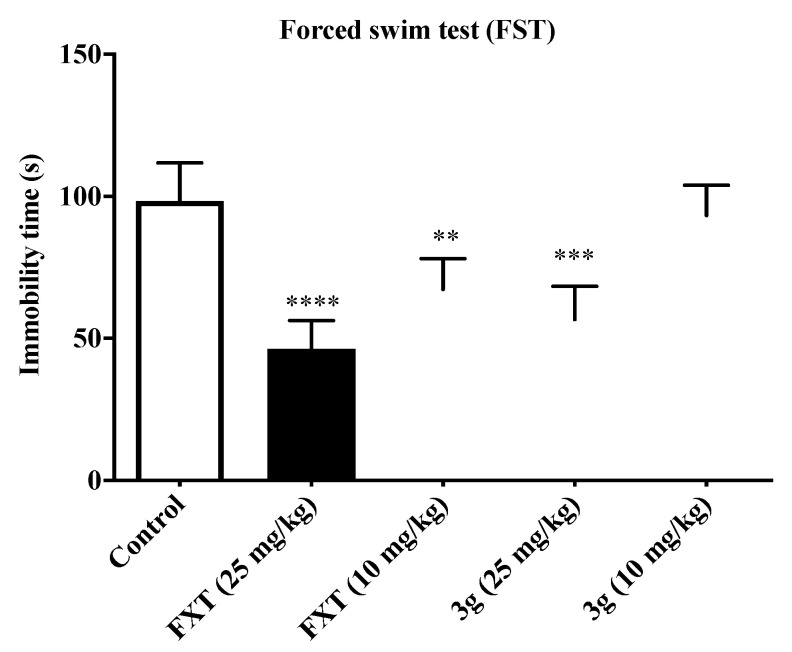
Effects of compound **3g** and FXT (i.p., 10 mg/kg and 25 mg/kg) on the duration of immobility time in the FST at lower dosages. Ten animals were used for each group. ** *p* < 0.01 compared to the control group, *** *p* < 0.001 compared to the control group, **** *p* < 0.001 compared to the control group.

Based on the performance of compound **3g** in the FST, its anti-immobility effect was evaluated at lower doses in the FST. As present in Figure 3, the compound **3g** and fluoxetine were effective in reducing the immobility time of mice at 25 mg/kg. Their antidepressive effects were dose-dependent. However, compound **3g** did not significantly affect the immobility time at the dosage of 10 mg/kg.

**Figure 4:**
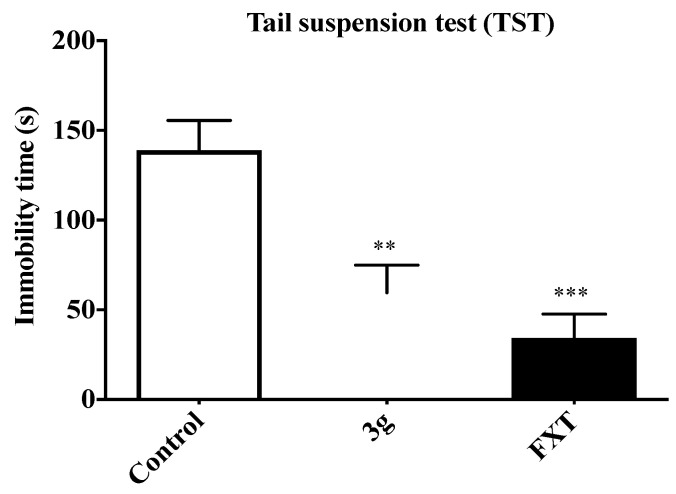
Effects of compound **3g** and FXT (i.p., 50 mg/kg) on the duration of immobility time in the TST. Ten animals were used for each group. ** *p* < 0.01 compared to the control group, *** *p* < 0.001 compared to the control group.

Apart from FST, a tail suspension test (TST) was conducted to further verify the antidepressant effect of **3g**. Compounds **3a** were i.p. administered with the dosage of 50 mg/kg. FXT (i.p., 50 mg/kg) used as the positive control. TST is also a widely used behavioral despair model for predicting the potential of an antidepressive candidate [35]. As shown in Figure 4, compound **3g** and FXT reduced immobility time significantly in the TST when taken at 50 mg/kg. This result further confirmed that compound **3g** has potent antidepressant activity.

**Figure 5:**
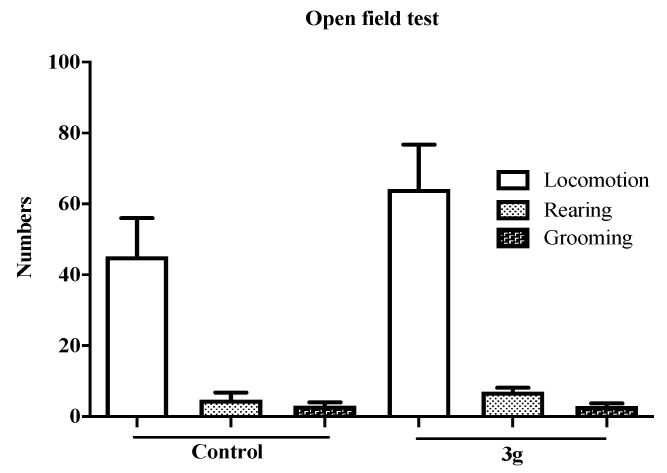
Effects of single treatment with **3g** (50 mg/kg) on the exploratory behaviors in the open-field test. Locomotion indicates the number of line crossings; rearing indicates the number of times seen standing on hind legs; grooming indicates the number of modifications. Ten animals were used for each group.

To exclude the possible false-positive of compound **3g** due to its effect on locomotor activity, the effect of compound **3g** on the locomotor activity of mice was assessed via an open-field test. The open-field test is a widely used behavioral test for the evaluation of the effects of drugs on autonomic activities and the exploratory behavior of animals [36]. As shown in Figure 5, compound **3g** showed no significant effect on locomotor activity. There was no significant difference in the crossing, rearing, and grooming of mice between the compound **3g** group and the control group. This result indicated that the anti-immobility activity of **3g** shown in the FST and TST is not led by CNS excitation.

### 2.3. Antiseizure Activity and Neurotoxicity

Both the maximal electroshock seizure (MES) and the subcutaneous pentylenetetrazole (sc-PTZ) model, widely used to screen antiseizure candidates in early drug discovery, were applied to determine the antiseizure effects of the target compounds (**3a**–**l**) [37]. Mice were treated i.p. with 30, 100, and 300 mg/kg of the test compounds and positive controls, and then tested at 0.5 and 4 h after administration. The protection of mice against the seizure was observed and recorded. Carbamazepine and valproate, currently used ASMs, were selected as the positive control and tested in the same situation [38].

As listed in Table 1, almost all molecules except **3k** displayed antiseizure activity in the MES model at 100 mg/kg or 300 mg/kg, while there was no compound that exhibited protection in the PTZ model even at 300 mg/kg. Compounds **3c**–**3d**, **3f**, and **3l** were found to be effective at 0.5 h intervals in the MES test. Furthermore, their antiseizure activity was also observed at 4h intervals when administrated at 300 mg/kg.

The CNS agents are usually accompanied by neurotoxicities such as slow reaction, dyskinesia, lethargy, and unclear consciousness. To assess the neurotoxicity of the target compounds, the rotarod test was carried out. As listed in Table 1, four compounds (**3c**, **3f**, **3d**, and **3l**) showed neurotoxicity at the maximum dose administered (300 mg/kg) in the rotarod test. At the dosage of 100 and 30 mg/kg, none of the compounds presented neurotoxicity in the rotarod test or gave any behavior related to nerve suppression or excitation such as mania, lethargy, and dyskinesia.

Compounds **3c**, **3f**, and **3g**, displaying antiseizure activity at 100 mg/kg in the MES test, were the three most active compounds. To obtain their accurate dose of antiseizure activity and neurotoxicity, they were re-subjected to the MES and rotarod tests. As listed in Table 2, compound **3c** had a median effective dose (ED_50_) value of 63.4 mg/kg and a median toxic dose (TD_50_) value of 264.1 mg/kg. Compound **3f** showed an ED_50_ value of 78.9 and a TD_50_ value of 253.5 mg/kg. Compound **3g** showed an ED_50_ value of 84.9 and a TD_50_ value of 439.9 mg/kg. All of them displayed higher anti-MES activity than valproate but lower activity than carbamazepine. PI value, a parameter used to evaluate the safety of antiseizure candidates, was calculated via dividing TD_50_ by ED_50_. As seen in Table 2, compounds **3c** and **3f** exhibited PI values of 4.2 and 3.2, which were lower than carbamazepine, but higher than valproate. Compound **3g** exhibited a superior PI value than carbamazepine and valproate (5.2 vs. 4.8 and 1.5).

### 2.4. Effects of Compound **3g** on the Level of Neurotransmitters GABA and 5-HT in Mouse Brain

The neurotransmitters (such as GABA, Glu, 5-HT, taurine, and so on), their corresponding receptors (such as GABAAR, GABABR, NMDA, AMPA, KAR, and so on), and ion channel (such as Cl^−^, Na^+^, K^+^, Ca^2+^) are the main targets or mechanisms involved in the current ASMs. In our previous study, antiseizure compounds containing triazole were confirmed to modulate GABAergic activity in mice [39,40]. With regard to depressive disorders, in addition to the monoamine hypothesis, other neurotransmitters, such as GABA and Glu, were also identified and confirmed to have a pathogenic role [41,42]. The GABA levels in occipital regions, ventromedial prefrontal regions, the dorsal anterolateral prefrontal, and the dorsomedial, were found to be downregulated in depression patients [43]. Based on the above, the effects of compound **3g** on the level of GABA and 5-HT in mice brains were investigated to verify the contribution of GABA and 5-HT in the antiseizure and antidepressant activities of it.

**Figure 6:**
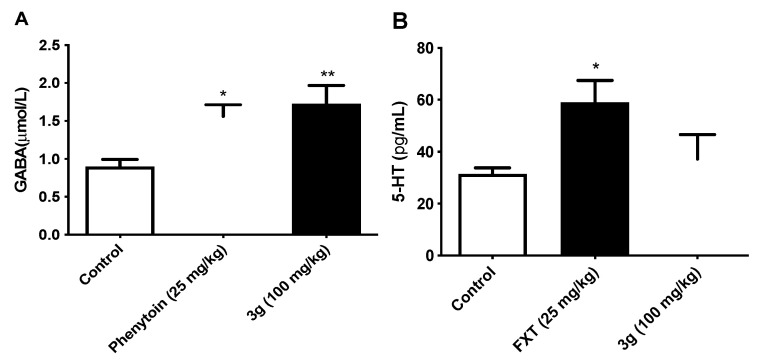
Effects of **3g**, phenytoin, and FXT on whole brain GABA (**A**) and 5-HT (**B**) levels in mice brains. Eight animals were used for each group. * *p* < 0.05 compared to the control group, ** *p* < 0.01 compared to the control group.

ELISA assays were carried out to explore the effects of **3g** on the level of GABA and 5-HT in mice brains. Phenytoin and FXT were used as the positive control [44,45]. As presented in Figure 6A, compounds **3g** and phenytoin improved the level of GABA in the mice brains significantly in comparation to the control group. FXT significantly increased the level of 5-HT in the mice brains nearly twice that of the control group. However, compound **3g** had no obvious effect on the level of **5-HT** in mice brain. The aforementioned suggested that the increasing of the GABA level in the brain might contribute to the antiseizure and anti-depressive effects of the compound **3g**.

To further confirm the above indication, the anti-MES and anti-immobility activity of compound **3g** was evaluated in the mice pretreated by thiosemicarbazide (TSC). As we know, GABA is synthesized by the decarboxylation of glutamate in the brain, and the reaction process is catalyzed by glutamate decarboxylase (GAD). TSC, as one of the GAD inhibitors, can interrupt the GABA synthesis, and reduce the level of GABA in the CNS [46].

**Figure 7:**
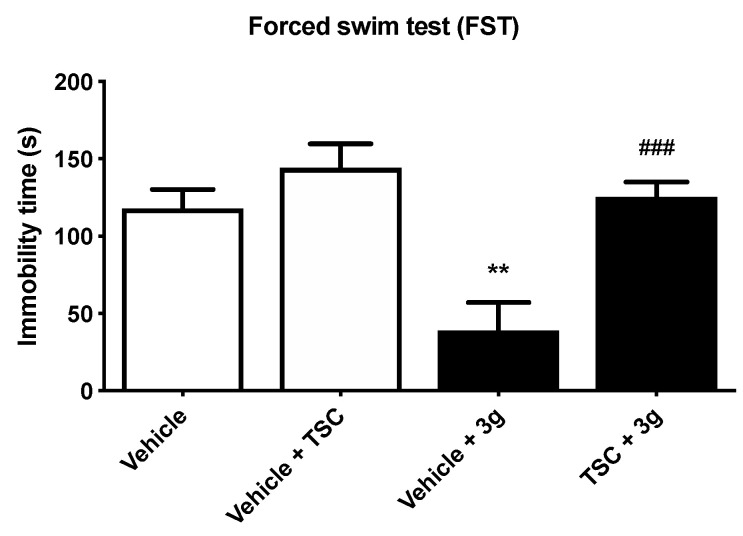
Effects of compound **3g** (50 mg/kg) on the immobility time in the TSC-treated mice in the FST. Eight animals were used for each group. ** *p* < 0.01 compared to the vehicle group; ^###^ *p* < 0.001 compared to the vehicle + **3g** group.

In this assay, mice were pretreated with 25 mg/kg of TSC (i.p.) for three consecutive days. Half an hour after the last administration, the treated mice were then subjected to the MES and FST. The results showed that the antiseizure activity disappeared in the MES test. No protection was obtained in the MES test when 100 or 300 mg/kg of compound **3g** (i.p.) was administered. In the FST, the anti-immobility activity of compound **3g** was also reversed. As shown in Figure 7, TSC did not affect the immobility time of mice when compared to the vehicle group. But the anti-immobility activity of compound **3g** at 50 mg/kg was reversed in the TSC-treated mice. The above results suggested that the upregulation of GABA levels in the brain of mice was involved in the antiseizure and antidepressant activities of compound **3g**.

As we mentioned previously, the common pathological basis of epilepsy and depression have been found. Some neurotransmitters such as GABA and 5-HT are associated with their common pathogenesis. In this study, the synthesized compound **3g** affected the GABA level, but not the 5-HT, which give it the potential to act as both an antiseizure and an antidepressant drug. The shared pathogenesis of epilepsy and depression make it possible to find some candidates for the depression and epilepsy comorbidities, such as compound **3g**.

### 2.5. Molecular Docking, Drug-Like Properties, and Pharmacokinetic Properties Prediction

**Figure 8:**
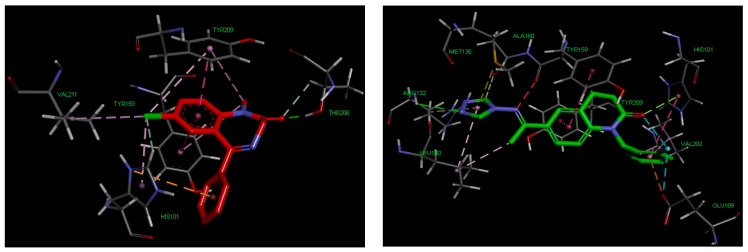
Interactions between compound **3g** (green), diazepam (red), and amino acid residues at the active site of the GABA_A_ receptor.

**Figure 9:**
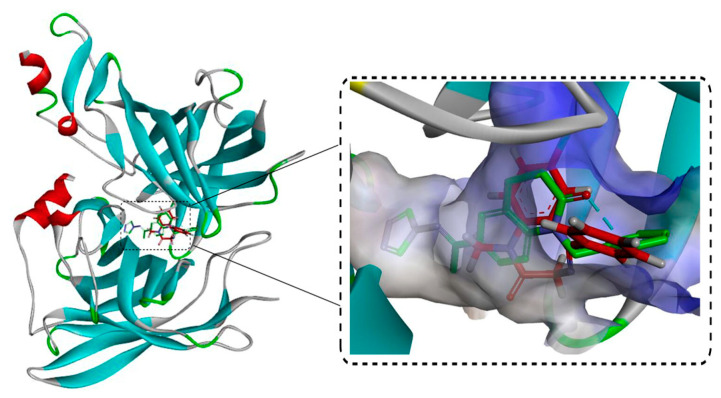
The overlying pattern of compound **3g** (green), and diazepam (red) in the binding pocket of the GABA_A_ receptor.

Based on the above finding, it is accepted that the GABAergic system was involved in the mechanism of action of compound **3g**. Molecular docking is a theoretical and computed method to study molecular interactions and predicts the mechanism of action. Therefore, a docking of compound **3g** and GABA_A_ receptor was performed to obtain the binding mode and molecular interactions. As seen in Figure 8, GABA_A_ agonist diazepam showed a hydrogen bond with Thr206; p–p stacking with TYR209 and HIS101; and a hydrophobicity interaction with TYR159 and VAL211. Compound **3g** showed a hydrogen bond with ARG132 and HIS101; p–alkyl with LEU140, ARG132, and VAL202, p–sulful with MET130; p–p stacking with TYR209 and TYR159; and a hydrophobicity interaction with GLU189, and VAL211. The overlying pattern of compound **3g** and diazepam was shown in Figure 9, which vividly presented that compound **3g** and diazepam had a similar binding model with the GABA_A_ receptor. Inspiringly, the docking scores for compound **3g** and the BZD-binding pocket was obtained to be 115.34, which is higher than that of diazepam, with a score of 104.52. These results suggested that compound **3g** might exert its pharmacologic effects by binding with the GABA_A_ receptor and have a similar mechanism of action to that of benzodiazepines.

Lipinski’s “Rule of Five” is widely used in early drug development such as drug design and screening. It suggests that most “drug-like” molecules have similar parameters, including LogP ≤ 5, molecular weight (MW) ≤ 500, the number of H-bond acceptors (HAB) ≤ 10, the number of H-bond donors (HBD) ≤ 5, and the number of rotatable bonds (ROTB) ≤ 10. Molecules that completely met the five rules indicate good oral bioavailability. As listed in Table 3, compounds **3a**–**l** met the Rule of Five, which indicates that they have a good drug-likeliness.

**Figure 10:**
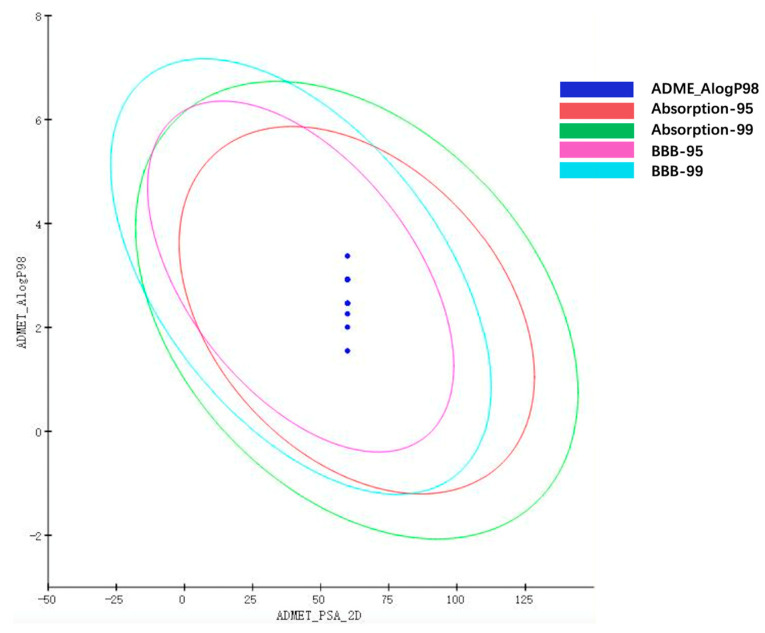
Prediction of ADME/T properties for compounds **3a**–**l**.

With regard to CNS agents, the absorption and blood–brain barrier permeability is especially important. To predict the pharmacokinetic properties of the molecules **3a**–**l**, a calculated molecular properties module in the DS 2019 platform was run to predict the absorption and blood–brain barrier permeability of the molecules **3a**–**l**. As illustrated in Figure 10, all compounds fell in the circle of absorption and BBB permeability, which indicated that they have favorable absorption and BBB permeability.

## 3. Materials and Methods

### 3.1. Chemiscal Part

The reagents used in this study were purchased from Macklin Inc. Thin-layer chromatography (TLC) was used to monitor the reaction progress. After purification, the products were sent to the analysis center for structure confirmation. NMR spectrums were measured on a Bruker AV-300 spectrometer. The HR-MS of compounds was measured on a Xevo G2-XS QT mass spectrometer.

#### 3.1.1. Synthesis Procedure of 6-Acetyl-3,4-dihydroquinolin-2(1H)-one (**1**)

3,4-Dihydro-1H-quinolinone (2.94 g, 20 mmol) and AlCl_3_ (9 g, 68 mmol) were mixed in CS_2_ solution (60 mL). Acetyl chloride (2.36g, 30 mmol) was added dropwise under the cooling of an ice bath. When the dropping is finished, the ice bath was removed, the mixture was reacted at 25 °C for 1h and then reacted at 55 °C for 24 h. When the reaction was completed, the reactant was dumped into ice water and the white sediment formed was filtered. The filter cake was purified by recrystallization with ethyl acetate. Compound **1** was obtained with a yield of 67%. Mp 165–167 °C. ^1^H-NMR δ ppm (CDCl_3_, 300 MHz): 2.57 (s, 3H, COCH_3_), 2.70 (t, *J* = 7.5 Hz, 2H, CH_2_), 3.04 (t, *J* = 7.5 Hz, 2H, CH_2_), 6.93 (d, *J* = 8.2 Hz, 1H, Ar-H), 7.79–7.83 (m, 2H, Ar-H), 9.80 (s, 1H, CONH). ^13^C NMR δ ppm (CDCl_3_, 75 MHz): 196.9, 141.6, 132.3, 128.6, 128.4, 128.3, 123.4, 115.4, 30.5, 26.4, 25.1. HR-MS (ESI) calculated for C_11_H_12_NO_2_^+^ ([M + H]^+^): 190.0863; measured: 190.0865.

#### 3.1.2. Synthesis Procedure of N-substituted-6-acetyl-3,4-dihydro-2(1H)-quinolinone (**2a**–**l**)

Using compound **2a** as an example: Compound **1** (0.60 g, 3.2 mmol) and NaOCH_3_ (0.70 g, 13 mmol) were mixed in a solution of CH_3_CN (20 mL). The mixture was stirred at 80 °C for 1h. After that, bromopropane (0.44 g, 3.6 mmol) was put into the flask and the reaction was raised to 120 °C for 15 h. The CH_3_CN was removed, and the residue was washed with water and filtered to get a crude product. It was purified by silica gel column chromatography using 2% CH_3_OH in CH_2_Cl_2_ as eluent. Yield: 77%. Mp: 78–80 °C, ^1^H-NMR δ ppm (CDCl_3_, 400 MHz): 0.97 (t, 3H, *J* = 7.5 Hz, Methyl), 1.64–1.71 (m, 2H, CH_2_), 2.58 (s, 3H, COCH_3_), 2.68 (t, *J* = 7.5 Hz, 2H, CH_2_), 2.96 (t, *J* = 7.5 Hz, 2H, CH_2_), 2.93 (t, *J* = 7.7 Hz, 2H, NCH_2_), 3.94 (t, *J* = 7.5 Hz, 2H, N-CH_2_), 7.03 (d, *J* = 8.5 Hz, 1H, Ar-H), 7.79 (s, 1H, Ar-H), 7.76 (d, *J* = 8.6 Hz, 1H, Ar-H). ^13^C NMR δ ppm (CDCl_3_, 126 MHz): 196.8, 170.1, 143.8, 131.6, 128.4, 128.1, 126.4, 114.5, 43.8, 31.6, 26.4, 25.5, 20.4, 11.2. HR-MS (ESI) calculated for C_14_H_18_NO_2_^+^ ([M + H]^+^): 232.1332; measured: 232.1337. Compounds **2b**–**l** were prepared by replacing bromopropane with other bromoalkanes or benzoyl chlorides according to the same procedure.

#### 3.1.3. Synthesis Procedure of Target Compounds (**3a**–**3l**)

4H-1,2,4-triazol-4-amine (1.04 g, 12 mmol), PTSA (0.12 g, 0.7 mmol), and compound **2** (2.9 mmol) were mixed with 12 mL toluene. The mixture, after stirring and refluxing overnight, was cooled under an ice bath. Finally, compound **3** was provided after the filtration, washing and recrystallization of the precipitated solid obtained above. The melting point, yield, and structural characterization data of the synthesized compounds (**3a**–**3l**) are shown below.

**(*E*)-6-(1-((4H-1,2,4-triazol-4-yl)imino)ethyl)-1-propyl-3,4-dihydroquinolin-2(1H)-one (3a)**: Mp 194–196 °C, yield 82%. ^1^H-NMR δ ppm (CDCl_3_, 300 MHz): 0.98 (t, *J* = 7.3 Hz, 3H, Methyl), 1.64–1.62 (m, 2H, -CH_2_), 2.37 (s, 3H, Methyl), 2.70 (t, 2H, *J* = 6.9 Hz, CH_2_), 2.98 (t, 2H, *J* = 6.9 Hz, CH_2_), 3.94 (t, 2H, *J* = 7.5 Hz, N-CH_2_), 7.01 (d, 1H, *J* = 9.3, Ar-H), 7.78–7.81 (m, 2H, Ar-H), 8.24 (s, 2H, Triazolo-H). ^13^C-NMR δ ppm (75 MHz, CDCl_3_): 172.25, 169.76, 143.06, 139.54, 129.06, 127.21, 126.98, 126.70, 114.73, 43.59, 31.44, 25.41, 20.24, 16.20, 11.06. HR-MS (ESI) calculated for C_16_H_20_N_5_O^+^ ([M + H]^+^): 298.1662; measured: 298.1659.

**(*E*)-6-(1-((4H-1,2,4-triazol-4-yl)imino)ethyl)-1-butyl-3,4-dihydroquinolin-2(1H)-one (3b)**: Mp 196–197 °C, yield 81%. ^1^H-NMR δ ppm (CDCl_3_, 300 MHz): 0.97 (t, 3H, *J* = 7.2 Hz, Methyl), 1.37–1.45 (m, 2H, CH_2_), 1.59–1.69 (m, 2H, CH_2_), 2.38 (s, 3H, Methyl), 2.70 (t, *J* = 6.7 Hz, 2H, CH_2_), 2.98 (t, *J* = 6.7 Hz, 2H, CH_2_), 3.98 (t, *J* = 7.6 Hz, 2H, N-CH_2_), 7.08–7.81 (m, 3H, Ar-H), 8.26 (s, 2H, Triazolo-H). ^13^C-NMR δ ppm (CDCl_3_, 75 MHz,): 172.32, 169.80, 143.18, 129.13, 127.31, 127.06, 126.83, 114.80, 41.95, 31.54, 29.63, 29.15, 25.51, 20.06, 16.27, 13.78. HR-MS (ESI) calculated for C_17_H_22_N_5_O^+^ ([M + H]^+^): 312.1819; measured: 312.1811.

**(*E*)-6-(1-((4H-1,2,4-triazol-4-yl)imino)ethyl)-1-pentyl-3,4-dihydroquinolin-2(1H)-one (3c)**: Mp 206–208 °C, yield 80%. ^1^H-NMR δ ppm (CDCl_3_, 300 MHz): 0.91 (t, *J* = 6.8 Hz, 3H, Methyl), 1.33–1.70 (m, 6H, CH_2_), 2.37 (s, 3H, Methyl), 2.69 (t, *J* = 6.8 Hz, 2H, CH_2_), 2.98 (t, *J* = 6.8 Hz, 2H, CH_2_), 3.96 (t, *J* = 7.6 Hz, 2H, N-CH_2_ ), 7.07 (d, *J* = 9.2 Hz, 1H, Ar-H), 7.79–7.82 (m, 2H, Ar-H), 8.26 (s, 2H, Triazolo-H). ^13^C-NMR δ ppm (CDCl_3_, 75 MHz): 172.26, 169.71, 143.07, 139.83, 129.03, 127.23, 126.98, 126.73, 114.70,, 42.10, 31.45, 28.85, 26.68, 25.41, 22.27, 16.19, 13.89. HR-MS (ESI) calculated for C_18_H_24_N_5_O^+^ ([M + H]^+^): 326.1975; measured: 326.1969.

**(*E*)-6-(1-((4H-1,2,4-triazol-4-yl)imino)ethyl)-1-hexyl-3,4-dihydroquinolin-2(1H)-one (3d)**: Mp 215–217 °C, yield 80%. ^1^H-NMR δ ppm (CDCl_3_, 300 MHz): 0.89 (t, *J* = 6.8 Hz, 3H, Methyl), 1.25–1.41 (m, 6H, CH_2_), 1.60–1.70 (m, 2H, CH_2_), 2.38 (s, 3H, Methyl), 2.70 (t, 2H, *J* = 6.7 Hz, CH_2_), 2.98 (t, *J* = 6.7 Hz, 2H, CH_2_), 3.96 (t, *J* = 7.7 Hz, 2H, N-CH_2_), 7.08 (d, *J* = 9.2 Hz, 1H, Ar-H), 7.80–7.83 (m, 2H, Ar-H), 8.25 (s, 2H, Triazolo-H). ^13^C-NMR δ ppm (CDCl_3_, 75 MHz): 172.32, 169.77, 143.17, 139.62, 129.13, 127.31, 127.06, 126.81, 114.79, 42.22, 31.54, 31.42, 27.03, 26.47, 25.51, 22.51, 16.27, 13.96. HR-MS (ESI) calculated for C_19_H_26_N_5_O^+^ ([M + H]^+^): 340.2132; measured: 340.2129.

**(*E*)-6-(1-((4H-1,2,4-triazol-4-yl)imino)ethyl)-1-heptyl-3,4-dihydroquinolin-2(1H)-one (3e)**: Mp 215–216 °C, yield 76%. ^1^H-NMR δ ppm (CDCl_3_, 300 MHz): 0.88 (t, *J* = 6.6 Hz, 3H, Methyl), 1.22–1.34 (m, 8H, CH_2_), 1.60–1.70 (m, 2H, CH_2_), 2.38 (s, 3H, Methyl), 2.67–2.72 (t, *J* = 6.7 Hz,2H, CH_2_), 2.98 (t, *J* = 6.7 Hz, 2H, CH_2_), 3.96 (t, *J* = 7.6 Hz, 2H, N-CH_2_), 7.08 (d, *J* = 9.2 Hz, 1H, Ar-H), 7.80–7.83 (m, 2H, Ar-H), 8.25 (s, 2H, Triazolo-H). ^13^C-NMR δ ppm (CDCl_3_, 75 MHz): 172.29, 169.73, 143.07, 139.57, 129.03, 127.22, 126.98, 126.72, 114.71, 42.15, 31.59, 31.44, 28.83, 26.99, 26.68, 25.41, 22.42, 16.18, 13.94. HR-MS (ESI) calculated for C_20_H_28_N_5_O ^+^ ([M + H]^+^): 354.2288; measured: 354.2277.

**(*E*)-6-(1-((4H-1,2,4-triazol-4-yl)imino)ethyl)-1-benzyl-3,4-dihydroquinolin-2(1H)-one (3f)**: Mp 246–247 °C, yield 74%. ^1^H-NMR δ ppm (CDCl_3_, 300 MHz): 2.33 (s, 3H, Methyl), 2.86 (t, *J* = 6.6 Hz, 2H, CH_2_), 3.08 (t, *J* = 6.6 Hz, 2H, CH_2_), 5.24 (s, 2H, CH_2_), 6.97 (d, *J* = 8.6 Hz, 1H, Ar-H), 7.19–7.35 (m, 5H, Ar-H), 7.64–7.67 (dd, *J* = 8.6, 1.9 Hz, 2H, Ar-H), 7.82 (d, *J* = 1.9 Hz, 1H, Ar-H), 8.24 (s, 2H, Triazolo-H). ^13^C-NMR δ ppm (CDCl_3_, 75 MHz): 172.12, 170.11, 143.17, 139.49, 136.16, 129.36, 128.81, 128.75, 127.23, 126.80, 126.60, 126.22, 115.54, 45.89, 31.42, 25.42, 16.15. HR-MS (ESI) calculated for C_20_H_20_N_5_O^+^ ([M + H]^+^): 346.1662; measured: 346.1660.

**(*E*)-6-(1-((4H-1,2,4-triazol-4-yl)imino)ethyl)-1-(2-fluorobenzyl)-3,4-dihydroquinolin-2(1H)-one (3g)**: Mp 201–202 °C, yield 82%. ^1^H-NMR δ ppm (CDCl_3_, 300 MHz): 2.35 (s, 3H, Methyl), 2.85 (t, *J* = 7.9 Hz, 2H, CH_2_), 3.08 (t, *J* = 7.9 Hz, 2H, CH_2_), 5.28 (s, 2H, CH_2_), 6.96 (d, *J* = 8.7 Hz, 1H, Ar-H), 7.02–7.28 (m, 4H, Ar-H), 7.67–7.70 (dd, *J* = 8.7, 2.1 Hz,2H, Ar-H), 7.82 (d, *J* = 2.1 Hz, 1H, Ar-H), 8.23 (s, 2H, Triazolo-H). ^13^C-NMR δ ppm (CDCl_3_, 75 MHz): 172.11, 170.21, 160.31 (d, ^1^*J*_c–f_ = 244.0 Hz), 142.77, 129.57, 128.94, 127.55 (d, ^3^*J*_c–f_ = 5.9 Hz), 127.37,126.88, 126.60, 124.39(d, ^4^*J*_c–f_ = 3.4 Hz), 123.10, 115.45(d, ^2^*J*_c–f_ = 14.0 Hz), 115.15, 39.58, 39.51, 31.42, 25.36, 16.20. HR-MS (ESI) calculated for C_20_H_19_FN_5_O^+^ ([M + H]^+^): 364.1568; measured: 364.1558.

**(*E*)-6-(1-((4H-1,2,4-triazol-4-yl)imino)ethyl)-1-(3-fluorobenzyl)-3,4-dihydroquinolin-2(1H)-one (3h)**: Mp 216–219 °C, yield 70%. ^1^H-NMR δ ppm (CDCl_3_, 300 MHz): 2.34 (s, 3H, Methyl), 2.85 (t, *J* = 8.0 Hz, 2H, CH_2_), 3.09 (t, *J* = 8.0 Hz, 2H, CH_2_), 5.22 (s, 2H, CH_2_), 6.87–7.03 (m, 4H, Ar-H), 7.28 (d, *J* = 8.6 Hz, 1H, Ar-H), 7.65–7.70 (dd, *J* = 8.6, 2.2 Hz, 2H, Ar-H), 7.82 (d, *J* = 2.2 Hz, 1H, Ar-H), 8.23 (s, 2H, Triazolo-H). ^13^C-NMR δ ppm (CDCl_3_, 75 MHz): 172.03, 170.08, 163.36 (d, ^1^*J*_c–f_ = 245.1 Hz), 142.87, 138.96, 138.87, 130.35, 129.61 (d, ^3^*J*_c–f_ = 8.2 Hz), 127.27, 126.94, 126.55, 121.88 (d, ^4^*J*_c–f_ = 1.8 Hz), 115.31, 114.21, 113.23 (d, ^2^*J*_c–f_ = 14.0 Hz), 45.47, 31.33, 25.35, 16.18. HR-MS (ESI) calculated for C_20_H_19_FN_5_O^+^ ([M + H]^+^): 364.1568; measured: 364.1560.

**(*E*)-6-(1-((4H-1,2,4-triazol-4-yl)imino)ethyl)-1-(4-fluorobenzyl)-3,4-dihydroquinolin-2(1H)-one (3i)**: Mp 290–292 °C, yield 71%. ^1^H-NMR δ ppm (CDCl_3_, 300 MHz): 2.33 (s, 3H, Methyl), 2.85 (t, *J* = 8.0 Hz, 2H, CH_2_), 3.07 (t, *J* = 8.0 Hz, 2H, CH_2_), 5.19 (s, 2H, CH_2_), 6.95 (d, *J* = 8.6 Hz, 1H, Ar-H), 6.9–7.03 (m, 2H, Ar-H), 7.17–7.21 (m, 2H, Ar-H), 7.66–7.69 (dd, *J* = 8.6, 2.2 Hz, 2H, Ar-H), 7.83 (d, *J* = 2.2 Hz, 1H, Ar-H), 8.25 (s, 2H, Triazolo-H). ^13^C-NMR δ ppm (CDCl_3_, 75 MHz): 172.50, 169.81, 161.16 (d, ^1^*J*_c–f_ = 237.8 Hz), 146.95, 142.19, 140.02, 133.08, 129.59, 128.48 (d, ^3^*J*_c–f_ = 8.3 Hz), 127.06 (d, ^3^*J*_c–f_ = 6.8 Hz), 126.67, 115.36, 115.33 (d, ^2^*J*_c–f_ = 21.8 Hz), 43.79, 30.95, 24.58, 16.18. HR-MS (ESI) calculated for C_20_H_19_FN_5_O^+^ ([M + H]^+^): 364.1568; measured: 364.1562.

**(*E*)-6-(1-((4H-1,2,4-triazol-4-yl)imino)ethyl)-1-(2-chlorobenzyl)-3,4-dihydroquinolin-2(1H)-one (3j)**: Mp 201–203 °C, yield 75%. ^1^H-NMR δ ppm (CDCl_3_, 300 MHz): 2.3 (s, 3H, Methyl), 2.87 (t, *J* = 8.0 Hz, 2H, CH_2_), 3.07 (t, *J* = 8.0 Hz, 2H, CH_2_), 5.28 (s, 2H, CH_2_), 6.80 (d, *J* = 8.7 Hz, 1H, Ar-H), 6.92 (dd, *J* = 8.6, 1.44 Hz, 1H, Ar-H), 7.12–7.26 (m, 2H, Ar-H), 7.42 (dd, *J* = 8.6, 1.4 Hz, 1H, Ar-H), 7.66 (dd, *J* = 8.7, 2.2 Hz, 2H, Ar-H ), 7.83 (d, *J* = 2.2 Hz, 1H, Ar-H), 8.26 (s, 2H, Triazolo-H). ^13^C-NMR δ ppm (CDCl_3_, 75 MHz): 172.05, 170.09, 142.81, 139.51, 133.03, 132.54, 129.68, 129.63, 128.44, 127.41, 127.05, 126.87, 126.58, 126.48, 115.32, 43.90, 31.40, 25.36, 16.20. HR-MS (ESI) calculated for C_20_H_19_ClN_5_O^+^ ([M + H]^+^): 380.1273; measured: 380.1270.

**(*E*)-6-(1-((4H-1,2,4-triazol-4-yl)imino)ethyl)-1-(3-chlorobenzyl)-3,4-dihydroquinolin-2(1H)-one (3k)**: Mp 216–219 °C, yield 80%. ^1^H-NMR δ ppm (CDCl_3_, 300 MHz): 2.34 (s, 3H, Methyl), 2.86 (t, *J* = 8.0 Hz, 2H, CH_2_), 3.08 (t, *J* = 8.0 Hz, 2H, CH_2_), 5.20 (s, 2H, CH_2_), 6.91 (d, *J* = 8.6 Hz, 1H, Ar-H), 7.08–7.01 (m, 1H, Ar-H) 7.12–7.26 (m, 2H, Ar-H), 7.18–7.26 (m, 3H, Ar-H), 7.64–7.68 (dd, *J* = 8.6, 2.1 Hz, 2H, Ar-H ), 7.84 (d, *J* = 2.1 Hz, 1H, Ar-H), 8.23 (s, 2H, Triazolo-H). ^13^C-NMR δ ppm (CDCl_3_, 75 MHz): 172.03, 170.09, 142.87, 139.50, 138.38, 134.70, 130.06, 129.62, 127.53, 127.32, 126.96, 126.58, 126.36, 124.45, 115.30, 45.46, 31.34, 25.36, 16.20. HR-MS (ESI) calculated for C_20_H_19_ClN_5_O^+^ ([M + H]^+^): 380.1273; measured: 380.1265.

**(*E*)-6-(1-((4H-1,2,4-triazol-4-yl)imino)ethyl)-1-(4-chlorobenzyl)-3,4-dihydroquinolin-2(1H)-one (3l)**: Mp 290–292 °C, yield 83%. 1H-NMR δ ppm (CDCl_3_, 300 MHz): 2.34 (s, 3H, Methyl), 2.85 (t, *J* = 7.9 Hz, 2H, CH_2_), 3.08 (t, *J* = 7.9 Hz, 2H, CH_2_), 5.20 (s, 2H, CH_2_), 6.92 (d, *J* = 8.6 Hz, 1H, Ar-H), 7.14–7.31 (m, 4H, Ar-H), 7.67 (dd, *J* = 8.6, 2.2 Hz, 2H, Ar-H), 7.84 (d, *J* = 2.2 Hz, 1H, Ar-H), 8.22 (s, 2H, Triazolo-H). ^13^C-NMR δ ppm (CDCl_3_, 75 MHz,): 171.78, 170.07, 139.47, 137.98, 132.38, 131.29, 129.01, 127.84, 127.30, 127.01, 124.60, 115.41, 111.65, 45.39, 31.46, 29.63, 25.50. HR-MS (ESI) calculated for C_20_H_19_ClN_5_O^+^ ([M + H]^+^): 380.1273; measured: 380.1268.

### 3.2. In Vivo Pharmacology

In this study, the antidepressant activity was determined through the FST and TST model. The locomotor activity was evaluated using the open-field test. The antiseizure activity were screened by MES and sc-PTZ. A rotarod test was used to evaluate the neurotoxicity. Kunming mice (20 ± 2 g) were used in all of the animal experiments. Polyethylene glycol-400 was used as the vehicle. The procedures involving animals were approved by the Medical Ethics Committee of Jinggangshan University (Approval No. 20200910). On the premise of obtaining reliable data, as few animals as possible were used. The detailed procedures of the mentioned tests were described in the previous articles [29,39,40]. In the determination of Brain GABA and 5-HT, mice (ten in each group) were administrated (i.p.) with the vehicle phenytoin (25 mg/kg), FXT (25 mg/kg), and compound **3g** (100 mg/kg), respectively. Once a day for three consecutive days, the mice were sacrificed by cervical dislocation. The brains of the mice were taken out, washed with cooling physiological saline, and homogenized (5000× *g*) in six volumes (g/mL) of physiological saline at 4 °C for 10 min. The supernatant was subjected to enzyme-linked immunosorbent assay (ELISA) kits (Biolegend, San Diego, CA, USA) to measure the content of the GABA and 5-HT in the mice brain. The level of GABA and 5-HT were indicated as μmol/L and pg/mL, respectively. The results were presented as means with standard error.

### 3.3. Molecular Docking, Drug-Like Properties, and Pharmacokinetic Properties Prediction

In the molecular docking study, the three-dimensional (3D) structure of the GABA_A_ receptor was downloaded from PDB (ID: 6 × 3x). The structure of the test molecule was constructed by ChemDraw 16.0 software, energy minimized, and docked with GABA_A_ using the docking module (LiDOCK) in Discovery Studio (release 2019). The binding mode of the ligand–receptor complex with the lowest energy was analyzed. In the prediction of drug-like properties and pharmacokinetic properties, ChemDraw Ultra 16.0 was used to construct the target compounds (**3a**–**l**). The calculate molecular properties module of DS 2019 was used to predict the drug-like properties and pharmacokinetic properties (i.e., MW, RotB, CLogP, nHBD, nHBA, absorption, and BBB permeability level) of the target compound (**3a**–**l**).

### 3.4. Statistical Analysis

The data from each group in the FST and TST were analyzed by one-way ANOVA, followed by Dunnet’s multiple comparison test. In the MES model, ED_50_ and TD_50_ values with their 95%CI were analyzed by log-probit analysis. For the comparation of ED_50_ value, the standard error (SEM) of the mean values were transformed from 95% confidence limits, and the ED_50_ with the SEM were compared using the one-way analysis of variance (ANOVA) followed by Dunnet’s test. One-way ANOVA, followed by Dunnet’s multiple comparison test was used for the comparison of the GABA and serotonin levels. All statistical analyses were performed with GraphPad Prism 7.0.

## 4. Conclusions

In this work, a dozen triazole-quinolinones (**3a**–**l**) were prepared and evaluated as newly CNS-active agents. All compounds displayed potent antidepressant activities in the FST. The majority of the target compounds displayed antiseizure action in the MES test. Compounds **3c**, **3f**, and **3g** displayed noticeable antidepressant activity in the FST and exhibited excellent antiseizure effects in the MES model with an ED_50_ of 63.4, 78.9, and 84.9 mg/kg, respectively. The effect of compound **3g** on the level of GABA in the mice brains has been proven to contribute to its antiseizure and antidepressant activities. The molecular docking study showed a good interaction between compound **3g** and the GABA_A_ receptor. The excellent drug-like properties and pharmacokinetic properties of compound **3a**–**l** were also predicted. These findings provided a new skeleton to develop agents for the treatment of epilepsy and depression comorbidities, although the antiseizure potency needs to be improved further and the mechanism of action needs further study. In the next step, a mouse comorbidity model of epilepsy and depression will be established to evaluate the antiseizure and antidepression activity of compound **3g**. In addition, we will further study its mechanism of action and continue to carry out structural modifications based on the analysis results of the mechanism research to obtain stronger and safer candidate drugs.

## Figures and Tables

**Table 1:** Antiseizure and neurotoxic effects of the target compounds (**3a**–**l**) after a single intraperitoneal injection in mice ^a^.

Compd.	-R	Maximal Electroshock Test		Pentylenetetrazole Test		Rotarod Test	
		0.5 h	4 h	0.5 h	4 h	0.5 h	4 h
**3a**	-C_3_H_7_	300	-	-	-	-	-
**3b**	-C_4_H_9_	300	-	-	-	-	-
**3c**	-C_5_H_11_	100	300	-	-	300	-
**3d**	-C_6_H_13_	300	300	-	-	300	-
**3e**	-C_7_H_15_	300	-	-	-	-	-
**3f**	-CH_2_C_6_H_5_	100	300	-	-	300	-
**3g**	-CH_2_C_6_H_4_(2-F)	100	-	-	-	-	-
**3h**	-CH_2_C_6_H_4_(3-F)	300	-	-	-	-	-
**3i**	-CH_2_C_6_H_4_(4-F)	300	-	-	-	-	-
**3j**	-CH_2_C_6_H_4_(2-Cl)	300	-	-	-	-	-
**3k**	-CH_2_C_6_H_4_(3-CI)	-	-	-	-	-	-
**3l**	-CH_2_C_6_H_4_(4-CI)	300	300	-	-	300	-
**Carbamazepine**	-	30	100	-	-	100	100
**Valproate**	-	300	-	300	-	-	-

^a^ Three mice were used for one dosage of a compound. The figure in the table represents the minimal dose in which antiseizure action was observed in two or three mice. The dotted line indicates that no antiseizure or neurotoxic effects were observed at the dose of 300 mg/kg.

**Table 2:** ED_50_ and TD_50_ values of **3c**, **3f**, and **3g** in the MES test.

Compounds	ED_50_ ^a^	TD_50_ ^b^	PI ^c^
**3c**	63.4 (58.0–69.3)	264.1 (240.1–290.5)	4.2
**3f**	78.9 (71.7–86.8)	253.5 (230.5–278.8)	3.2
**3g**	84.9 (75.16–95.83)	439.9 (394.45–490.70)	5.2
**Carbamazepine**	8.7 (8.3–10.2)	41.5 (38.1–46.3)	4.8
**Valproate**	288 (257–329)	432 (370–492)	1.5

^a^ ED_50_—median effective dose required to assure antiseizure protection in 50% animals. ^b^ TD_50_—median toxic dose eliciting minimal neurological toxicity in 50% animals. ^c^ PI—protective index (TD_50_/ED_50_).

**Table 3:** Drug-like property parameters of the target compounds **3a**–**l**.

Compound	MW	CLogP	HBD	HBA	n-ROTB	Lipinski’s Violation
**Rule**	500	≤5	≤5	<10	≤10	≤1
**3a**	297.355	1.549	0	4	4	0
**3b**	311.382	2.005	0	4	5	0
**3c**	325.408	2.462	0	4	6	0
**3d**	339.435	2.918	0	4	7	0
**3e**	353.461	3.374	0	4	8	0
**3f**	345.398	2.26	0	4	4	0
**3g**	363.388	2.466	0	4	4	0
**3h**	363.388	2.466	0	4	4	0
**3i**	363.388	2.466	0	4	4	0
**3j**	379.843	2.925	0	4	4	0
**3k**	379.843	2.925	0	4	4	0
**3l**	379.843	2.925	0	4	4	0

## Data Availability

Not applicable.

## Sample Availability

Samples of the compounds are available from the authors.
